# Real-world data on neoadjuvant chemotherapy with dual-anti HER2 therapy in HER2 positive breast cancer

**DOI:** 10.1186/s12885-024-11871-0

**Published:** 2024-01-25

**Authors:** Zheng-Jun Yang, Fei Xin, Zu-Jin Chen, Yue Yu, Xin Wang, Xu-Chen Cao

**Affiliations:** 1 The First Department of Breast Cancer, Tianjin Medical University Cancer Institute and Hospital, National Clinical Research Center for Cancer, Huan-Hu-Xi Road, He-Xi District, Tianjin, 300060 China; 2 Key Laboratory of Cancer Prevention and Therapy, Tianjin, 300060 China; 3 Key Laboratory of Breast Cancer Prevention and Therapy, Tianjin Medical University, Ministry of Education, Tianjin, 300060 China; 4 Tianjin’s Clinical Research Center for Cancer, Tianjin, 300060 China

**Keywords:** HER2 positive, Breast cancer, Neoadjuvant chemotherapy, Dual-targeted therapy

## Abstract

**Background:**

Neoadjuvant chemotherapy with dual-targeted therapy is the standard treatment for human epidermal growth factor 2 (HER2)-positive breast cancer. Although the dual-targeted therapy has significantly improved the pathological complete response (pCR) rate, further investigation is needed to identify biomarkers that predict the response to neoadjuvant therapy.

**Methods:**

This retrospective study analyzed 353 patients with HER2-positive breast invasive ductal carcinoma. The correlation between clinicopathological factors and pCR rate was evaluated. A nomogram was constructed based on the results of the multivariate logistic regression analysis to predict the probability of pCR.

**Results:**

The breast pCR (b-pCR) rate was 56.1% (198/353) and the total pCR (t-pCR) rate was 52.7% (186/353). Multivariate analysis identified ER status, PR status, HER2 status, Ki-67 index, and neoadjuvant chemotherapy regimens as independent indicators for both b-pCR and t-pCR. The nomogram had an area under the receiver operating characteristic curve (AUC) of 0.73 (95% CI: 0.68–0.78). According to the nomogram, the t- pCR rate was highest in the ER-PR- HER2-positive patients (131/208) and lowest in the ER + PR + HER2-positive patients (19/73). The subgroup analyses showed that there was no significant difference in pCR rate among the neoadjuvant chemotherapy regimens in ER positive, PR positive, HER2 IHC 2 + , Ki67 index < 30% population. However, for ER-PR-HER2-positive patients, the neoadjuvant chemotherapy regimen has a great influence on the pCR rates.

**Conclusions:**

Patients with ER-negative, PR-negative, HER2 3 + and high KI-67 index were more likely to achieve pCR. THP may be used as an alternative to AC-THP or TCbHP in selected HER2-positive patients.

**Supplementary Information:**

The online version contains supplementary material available at 10.1186/s12885-024-11871-0.

## Introduction

As is well-known, the introduction of pertuzumab has significantly improved the survival outcomes of patients with HER2-positve breast cancer. Neoadjuvant chemotherapy with dual anti-HER2 therapy by trastuzumab and pertuzumab has become the standard therapy for local advanced HER2-positive breast cancer. Despite its approval for anti-HER2 therapy in 2019 in our country, pertuzumab is not widely used in neoadjuvant therapy due to its relatively higher price. Real word data on neoadjuvant chemotherapy with trastuzumab and pertuzumab is still limited. Achieving pCR is a critical goal of neoadjuvant therapy. Due to the limited clinical data, the biomarkers for predicting response to neoadjuvant chemotherapy with dual anti-HER2 therapy remain unclear and require further investigation. Our study included 353 patients with HER2-positive breast cancer who received neoadjuvant chemotherapy with trastuzumab and pertuzumab. The chemotherapy regimens used in our study were six cycles docetaxel and carboplatin with trastuzumab and pertuzumab (TCbHP), four cycles docetaxel with trastuzumab and pertuzumab (THP) and four cycles anthracyclines and cyclophosphamide sequential four cycles docetaxel with trastuzumab and pertuzumab (AC-THP).

Our previous study confirmed that PR expression was significantly associated with the survival outcome and pCR in hormone receptor positive and HER2-negative breast cancer [1]. However, the use of PR expression level as a biomarker for predicting pCR in HER2-positive breast cancer patients is still controversial [2–4]. According to the guidelines, HER2 immunohistochemistry 2 + with positive amplification through fluorescence in situ hybridization can be treated with anti-HER2 therapy. Recent studies have shown a positive correlation between HER2 copy number and pCR, indicating a higher HER2 copy number are more likely to achieve pCR [4, 5]. Therefore, further investigation is needed to determine if a significant difference exists between HER2 2 + and HER2 3 + in pCR rates. In our clinical practice, TCbHP and AC-THP are the most used regimens in neoadjuvant therapy, which can dramatically improve pCR rate but have a high occurrence of side effects. During the COVID-19 period, patients were less likely to receive neoadjuvant chemotherapy due to concerns that chemotherapy may increase the risk of COVID-19-related complications [6]. To address this problem, de-escalation of neoadjuvant chemotherapy regimens has been proposed. THP has gained popularity in recent years particularly during the COVID-19 period, due to its low incidence of grade 3–4 level toxicities compared with TCbHP or AC-THP. However, studies focus on THP in neoadjuvant treatment are still insufficient to determine whether THP regimen can be widely used in HER2-positive breast cancer patients. A predictive nomogram based on combined clinicopathological factors is urgently to identify response to neoadjuvant chemotherapy with trastuzumab and pertuzumab.

In summary, previous studies showed that Ki67 index, ER expression, PR expression and HER2 expression may be closely associated with pCR. However, relying on a single clinicopathological factor among these above-mentioned predictive factors is not sufficient to accurately identify a patient’s response to neoadjuvant chemotherapy with trastuzumab and pertuzumab. Thus, there is an urgent need to construct a nomogram using combined clinicopathological factors. Additionally, we aimed to evaluate the predictive efficacy of a de-escalated chemotherapy with trastuzumab and pertuzumab in selected HER2-positive breast cancer, and to determine whether the THP regimen can be used as an alternative to TCbHP or AC-THP in such cases.

## Patients and methods

### Patients

We conducted a retrospective analysis of 353 patients diagnosed with HER2-positive invasive ductal breast cancer who received neoadjuvant chemotherapy with trastuzumab and pertuzumab in the Tianjin Medical University Cancer Institute and Hospital from May 2019 to December 2022. All patients underwent surgery and had no prior history of cancer or bilateral tumors. Informed consent was obtained from all patients, and the the research protocol was approved by the Ethics Committees at the Tianjin Medical University Cancer Institute and Hospital. The cutoff value for ER and PR positive was set at 10% [7]. HER2-positive defines as immunohistochemistry (IHC) 3 + or IHC 2 + with positive amplification through fluorescence in situ hybridization.

### Statistical analysis

Statistical analysis was conducted by presenting the general characteristics of study subjects as mean ± standard deviation (SD) for continuous variables and the number (percentage) for categorical variables. Paired t-test was used for numerical variables and chi-square or Fisher’s exact test for categorical data. Multivariate logistic regression was used to obtain the odds ratio (OR) with 95% confidence interval (CI) for the association with the pCR. A nomogram was constructed based on the results of the multivariate logistic regression analysis to predict the probability of pCR. ROC curve analysis was used to assess the prediction power of the nomogram. For all analyses, a *P*-value < 0.05 was considered significant. All statistical analyses were performed using R software version 4.04 (The R Foundation for Statistical Computing).

### Evaluation of pathological response

Total pCR defined as total pathological complete response in the breast and lymph nodes (ypT0/isypN0, absence of invasive cancer in the breast and axillary lymph nodes, regardless of the remaining ductal carcinoma in situ in the primary tumor). b-PCR defines as breast pathological complete response (ypT0/is).

### Scheme of treatment

All patients included in this study received neoadjuvant chemotherapy with dual anti-HER2 therapy according to the guidelines. The treatment regimens consist of six cycles docetaxel and carboplatin with trastuzumab and pertuzumab (TCbHP), four cycles of docetaxel with trastuzumab and pertuzumab (THP) and four cycles anthracyclines and cyclophosphamide followed by four cycles docetaxel with trastuzumab and pertuzumab (AC-THP). Surgery was performed three to four weeks after the completion of neoadjuvant therapy.

## Results

A total of 353 patients with HER2-positive invasive ductal breast cancer who received neoadjuvant chemotherapy with trastuzumab and pertuzumab therapy were included. The clinicopathological features and treatment modalities were summarized in Tables 1 and 2. Among the patients, 236 of 353 patients (66.9%) with clinical T1-2 stage diseases and 117 (33.1%) had tumor stage T3-4. The number of patients with clinical lymph node negative was 58 (16.4%), and 83.6% with clinical lymph node positive disease. A total of 37 patients received AC-THP therapy (10.5%), 60.3% received TCbHP therapy, and 29.2% received THP therapy. A total of 200 patients were ER-negative (56.7%), 79.3% were PR-negative, 84.1% were HER2 IHC 3 + , and 91.5% had a Ki67 index of ≥ 30%.

**Table 1:** Patient characteristics according to total pathological complete response

	Non-pCR (*n* = 168)	pCR (*n* = 185)	All (*n* = 353)	*P*-value
Age (years), mean ± SD	49.39 ± 10.20	48.54 ± 9.92	48.94 ± 10.04	0.427
Tumor stage				
cT1-2	107	129	236	0.320
cT3-4	61	56	117	
Lymph node status				
Negative	23	35	58	0.186
Positive	145	150	295	
ER status				
Negative	72	128	200	< 0.001
Positive	96	57	153	
PR status				
Negative	114	166	280	< 0.001
Positive	54	19	73	
Histological Grade				
I-II	97	92	189	0.132
III	71	93	164	
Ki-67 index				
< 30%	21	9	30	0.010
≥ 30%	147	176	323	
HER2 status				
2 +	43	13	56	< 0.001
3 +	125	172	297	
IMPC				
With	22	0	22	< 0.001
Without	146	185	331	
Chemotherapy regimens				
THP*4	57	46	103	0.077
TCbHP*6	91	122	213	
AC*4-THP*4	20	17	37

**Table 2:** Patient characteristics according to breast pathological complete response

	Non-pCR (*n* = 155)	pCR (*n* = 198)	All (*n* = 353)	*P*-value
Age (years), mean ± SD	49.38 ± 10.19	48.53 ± 9.91	48.94 ± 10.04	0.295
Tumor stage				
cT1-2	100	136	236	0.493
cT3-4	55	62	117	
Lymph node status				
Negative	23	35	58	0.475
Positive	132	163	295	
ER status				
Negative	63	137	200	< 0.001
Positive	92	61	153	
PR status				
Negative	104	176	280	< 0.001
Positive	51	22	73	
Histological Grade				
I-II	94	95	189	
III	61	103	164	0.018
Ki-67 index				
< 30%	20	10	30	0.009
≥ 30%	135	188	323	
HER2 status				
2 +	42	14	56	< 0.001
3 +	113	184	297	
IMPC				
with	20	2	22	< 0.001
without	135	196	331	
Chemotherapy regimens				
THP*4	49	54	103	0.332
TCbHP*6	87	126	213	
AC*4-THP*4	19	18	37	

As shown in Tables 1 and 2,overall breast pCR rate was 56.1% (198/353) and the total pCR rate was 52.4% (185/353). As shown in Tables 3 and 4, the multivariate analyses revealed that ER expression, PR expression, Ki67 index, and HER2 status were independent predictors of pCR. Interestingly, our association analysis showed that none of patients with mixed invasive micropapillary carcinoma achieved total pCR, although this result requires further investigation.

**Table 3:** Multivariate logistic regression models predicting total pathological complete response

Variables	Coefficient	OR (95% CI)	*P*-value
Age	-0.020	0.980 (0.957,1.004)	0.108
Tumor stage			
cT1-2		Reference	
cT3-4	-0.362	0.697 (0.422,1.150)	0.158
Lymph node status			
Negative		Reference	
Positive	-0.455	0.634 (0.330,1.221)	0.173
ER status			
Negative		Reference	
Positive	-0.829	0.437 (0.241,0.789)	0.006
PR status			
Negative		Reference	
Positive	-1.077	0.341 (0.160,0.724)	0.005
Ki-67 index			
< 30%		Reference	
≥ 30%	1.225	3.404 (1.357,8.538)	0.009
HER2 status			
2 +		Reference	
3 +	1.326	3.765 (1.776,7.985)	< 0.001
Chemotherapy regimens			
THP*4		Reference	
TCbHP*6	1.005	2.733 (1.562,4.782)	< 0.001
AC*4-THP*4	1.138	3.121 (1.257,7.748)	0.014

Logistic regression prediction model: $\pi (Y=1)=\frac{1}{1+\mathit{exp}(-Score)}$

$$\begin{array}{l}score=-0.829\left(\text{ER}=\text{Positive}\right)-1.077\left(\text{PR}=\text{Positive}\right)+1.225(\text{Ki}-67\\\geq30\%)+1.326\left(\text{HER}2=3+\right)+1.005(\mathrm{chemotherapy}\;\mathrm{regimen}=\text{TCbHP}\ast6)\\+1.138(\mathrm{chemotherapy}\;\mathrm{regimen}=\text{AC}\ast4-\text{THP}\ast4)\end{array}$$

**Table 4:** Multivariate logistic regression models predicting breast pathological complete response

	Coefficient	OR (95% CI)	*P*-value
Age	-0.026	0.974 (0.950,0.999)	0.044
Tumor stage			
cT1-2		Reference	
cT3-4	-0.261	0.771 (0.459,1.294)	0.324
Lymph node status			
Negative		Reference	
Positive	-0.173	0.841 (0.431,1.638)	0.610
ER status			
Negative		Reference	
Positive	-0.996	0.370 (0.201,0.680)	0.001
PR status			
Negative		Reference	
Positive	-0.834	0.434 (0.205,0.921)	0.030
Ki-67 index			
< 30%		Reference	
≥ 30%	1.240	3.457 (1.347,8.700)	0.008
HER2 status			
2 +		Reference	
3 +	1.353	3.868 (1.828,8.184)	< 0.001
IMPC			
Without		Reference	
With	-2.744	0.064 (0.014,0.299)	< 0.001
Chemotherapy regimens			
THP*4		Reference	
TCbHP*6	0.699	2.012 (1.131,3.577)	0.017
AC*4-THP*4	1.024	2.785 (1.080,7.182)	0.034

Logistic regression prediction model: $\pi (Y=1)=\frac{1}{1+\mathit{exp}(-Score)}$

$$\begin{array}{c}score=-0.996\left(\text{ER}=\text{Positive}\right)-0.834\left(\text{PR}=\text{Positive}\right)+1.240(\text{Ki}-67\\\geq30\%)+1.353\left(\text{HER}2=3+\right)-2.744(\text{IMPC}\\=\text{with})+0.699(\mathrm{chemotherapy}\;\mathrm{regimen}\\=\text{TCbHP}\ast6)+1.024(\mathrm{chemotherapy}\;\mathrm{regimen}=\text{AC}\ast4-\text{THP}\ast4)\end{array}$$

**Fig. 1:**
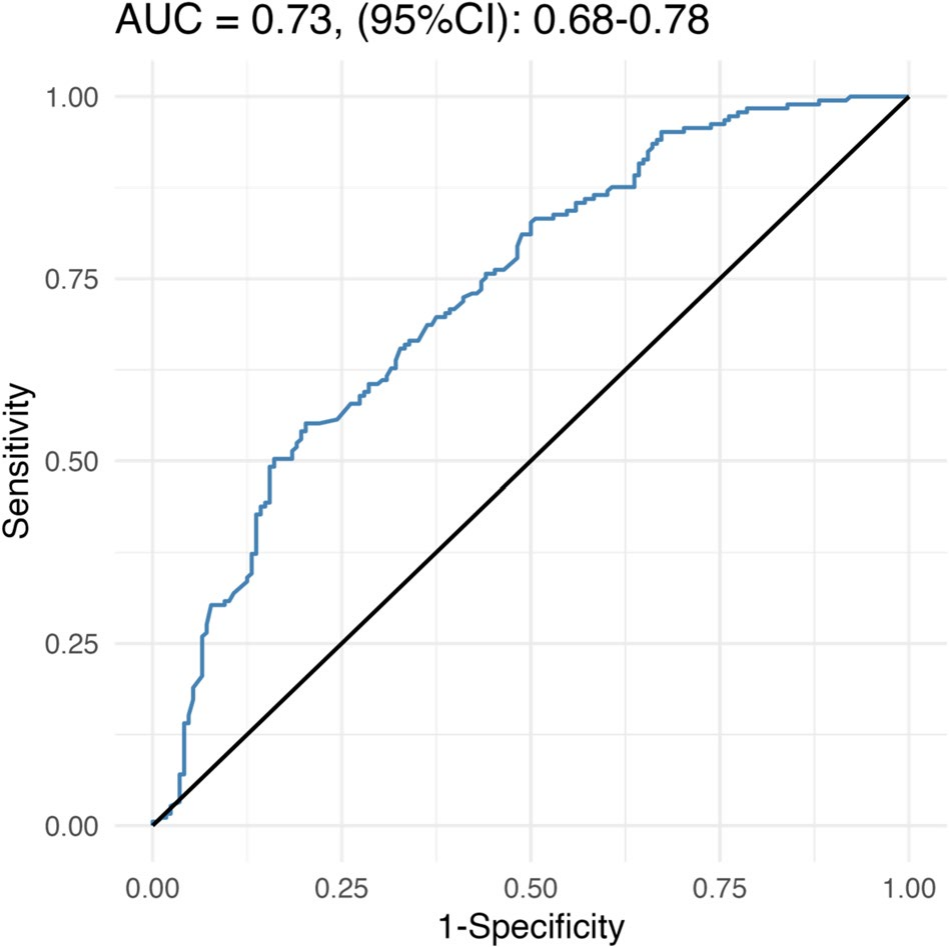
The Receiver operating characteristic (ROC) curve of the nomogram for predictability of total pCR in HER2 breast cancer patients

**Fig. 2:**
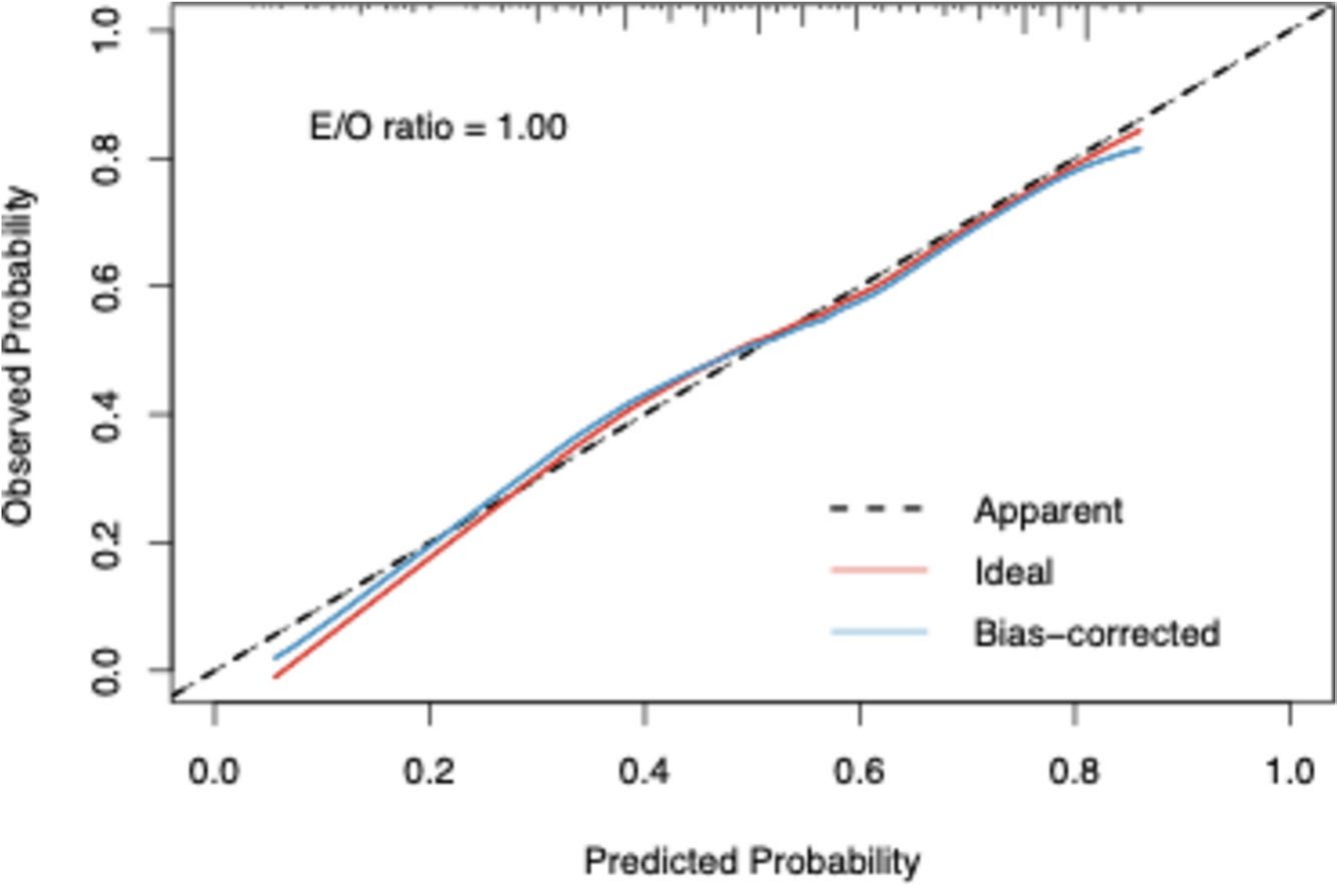
Calibration curve of the nomogram in the whole set of HER2 breast cancer patients

Based on these results, we constructed a nomogram to predict pCR (Tables 3 and 4). Using this nomogram model, the AUC value was 0.73 (95% CI: 0.68–0.78) (Fig. 1). The performance of the nomogram was validated with a calibration curve, which showed good agreement between the actual observations and the predicted outcomes in the whole set. The prediction curve was close to the standard curve (Y = X), indicating that the model had good performance and high application (Fig. 2). As shown in Table S4, the sensitivity is 71.89% and the specificity is 73.21%, indicating that the predictive ability of this nomogram needs further improvement.

As shown in Tables 5 and 6, subgroup analyses demonstrated that there was no significant difference in pCR rate among the neoadjuvant chemotherapy regimens in the population with ER-positive, PR-positive, HER2 IHC 2 + , and Ki67 index < 30%. However, for ER-PR-HER2-positive patients, the neoadjuvant chemotherapy regimen has a great influence on the pCR rates (Tables 7 and 8).

**Table 5:** Associations between chemotherapy regimen and t-pCR in different subgroups

Combined group	THP	Stratification by chemotherapy regimens	AC-THP
		TCbHP	
ER Status			
ER-Negative	Ref	2.521 (1.266–5.022)*	1.867 (0.504–6.915)
ER-Positive	Ref	1.136 (0.384–3.365)	1.828 (0.429–7.791)
PR Status			
PR-Negative	Ref	2.194 (1.184–4.065)*	1.791 (0.691–5.185)
PR-Positive	Ref	1.009 (0.172–7.036)	2.816 (0.332–23.859)
HER2 Status			
2 +	Ref	0.395 (0.052–2.978)	0.485 (0.065–3.628)
3 +	Ref	2.307 (1.259–4.226)*	3.267 (1.051–10.158)*
Ki-67 index			
< 30%	Ref	0.701 (0.131–3.738)	0.745 (0.057–9.702)
≥ 30%	Ref	2.215 (1.233–3.979)*	3.927 (1.381–11.172)*

^*^represents *p* < 0.05

**Table 6:** Associations between chemotherapy regimen and b-pCR in different subgroups

Combined group	THP	Stratification by chemotherapy regimens	AC-THP
		TCbHP	
ER Status			
ER-Negative	Ref	2.916 (1.539–5.527)*	2.238 (0.652–7.679)
ER-Positive	Ref	1.929 (0.608–5.527)	2.307 (0.516–10.307)
PR Status			
PR-Negative	Ref	2.500 (1.401–4.461)*	2.123 (0.772–5.842)
PR-Positive	Ref	3.019 (0.299–30.424)	4.809 (0.364–63.564)
HER2 Status			
2 +	Ref	0.766 (0.101–5.810)	0.842 (0.111–6.382)
3 +	Ref	2.697 (1.512–4.812)*	2.771 (0.978–7.857)
Ki-67 index			
< 30%	Ref	0.918 (0.156–5.389)	0.722 (0.053–9.810)
≥ 30%	Ref	2.672 (1.521–4.695)*	3.544 (1.353–9.339)*

^*^represents *p* < 0.05

**Table 7:** Associations between chemotherapy regimen and tpCR in different combined groups

Combined group	Total	Stratification by tpCR		
	*n* = 353	No	Yes	*P* Value
ER + PR + HER2-positive	73	54	19	0.102
AC-TPH		9	4	
TCbPH		34	15	
TPH		11	0	
ER + PR-HER2-positive	72	36	36	0.850
AC-TPH		4	3	
TCbPH		27	29	
TPH		5	4	
ER-PR-HER2-positive	208	77	131	0.014
AC-TPH		7	10	
TCbPH		30	78	
TPH		40	43

**Table 8:** Associations between chemotherapy regimen and b-pCR in different combined groups

Combined group	Total	Stratification by b-pCR		
	*n* = 353	No	Yes	*P* Value
ER + PR + HER2-positive	73	51	22	0.554
AC-TPH		8	5	
TCbPH		34	15	
TPH		9	2	
ER + PR-HER2-positive	72	36	36	0.850
AC-TPH		4	3	
TCbPH		27	29	
TPH		5	4	
ER-PR-HER2-positive	208	68	140	0.022
AC-TPH		7	10	
TCbPH		26	82	
TPH		35	48	

## Discussion

Chemotherapy combined with anti-HER2 therapy remains the standard treatment for HER2 positive breast cancer. Due to the limited clinical data, the biomarkers for predicting response to neoadjuvant chemotherapy with dual anti-HER2 therapy remain unclear and require further investigation. In our present study, multivariate analyses revealed that PR expression, Ki67 index, and HER2 status were independent predictors of pCR. Based on these results, we constructed a nomogram to predict pCR. According to the nomogram, we found that patients with PR negative, higher Ki67 index and HER2 3 + are more likely to achieve pCR when undergone neoadjuvant chemotherapy with dual anti-HER2 therapy. The significance of this nomogram can predict the sensitivity of patients to neoadjuvant chemotherapy combined with targeted therapy in the future, thus selecting a more suitable treatment plan. On the premise of ensuring treatment effectiveness, try to choose a de-escalated treatment plan to reduce adverse reactions and toxic side effects of patients, and improve their compliance and tolerance. Thus, achieving precise personalized treatment.

Consistent with previous study [8], our present study also showed that pCR rates vary according to ER/PR status, with the highest rates observed in ER-PR-HER2-positive breast cancer patients and the lowest in the ER + PR + HER2-positive breast cancer patients. Patients with ER + PR + HER2 positive (triple positive breast cancer) who received the standard neoadjuvant therapy still had the lowest pCR rates. To address this issue, we must first identify the reasons for the difference in pCR rates between HR-HER2-positive and HR + HER2-positive patients.

Firstly, HER2 positive disease is clinically and biologically heterogenous and not all patients benefit equally from the current therapies. Previous studies showed that HER2 disease was biologically heterogeneous and encompassed a spectrum of distinct molecular subtypes (Luminal A, Luminal B, HER2-enriched and basal-like) [9–12]. A recently published paper showed that HER2 heterogeneity is the most frequent in HR + HER2-positive disease with an incidence of 10% and is associated with lower pCR rates [13]. However, the current definition of HER2 positive does not sufficiently consider the heterogeneity of HER2-positive disease.

Secondly, as well known, the HER2-enriched subtype is more likely to activate the HER2 pathway and benefit the most from dual anti-HER2 therapy. However, there is a significantly difference in the distribution of HER2-enriched subtype between HR + HER2-positive and HR-HER2-positive (54% vs 81%). Although there was a clear relationship between HER2-enriched subtype and ERBB2 levels, they still provide additional information from each other. So, HER2-enriched subtype and ERBB2 levels should not be considered the same, and their combination into a single variable has a better predictive value for the rate of pCR and the pCR rate was highest in the HER2-enriched/ERBB2-high subtype, which represented 68.1% of HR-HER2-positive disease. However, this proportion significantly decreased to 31.7% in HR + HER2-positive disease [14]. Thus, the difference in distribution between HR-HER2-positive and HR + HER2-positive disease may be one reason why the pCR rate was significantly lower in HR + HER2-positive (especially in triple positive) than HR-HER2-positive breast cancer patients.

Thirdly, the drivers of HR + HER2-positive may differ from those HR-HER2-positive cancers. Wang et al. reported that some HR + HER2-positive breast cancer cells might be primarily driven by the ER pathway and weakly driven by the HER2 pathway, making them intrinsically less sensitive to anti-HER2 treatment and possible sensitive to endocrine therapy [15]. This may be another reason why pCR rates were lower in HR + HER2-positive than HR- HER2-positive breast cancer patients. Additionally, there is a complex molecular signaling crosstalk between the ER/PR and HER2 pathways, which may contribute to the low sensitivity to neoadjuvant chemotherapy with dual anti-HER2 therapy in HR + HER2-positive patients [16].

Therefore, to improve the pCR rates of the triple positive subgroup, we should take HER2 heterogeneity and ER/PR status into consideration and not merely rely on the routine use of HER2 definition [13]. One strategy to improve the pCR rates is to combine endocrine therapy with anti-HER2 therapy simultaneously block both ER and HER2 signaling pathways. The MUKDEN 01 trail showed that triple positive breast cancer patients who received CDK4/6i + AI combined with anti HER2 therapy achieved a pCR rate of 30.4%, suggesting that a selected subgroup of HR + HER2-positive patients may benefit from this combined therapy without chemotherapy in the neoadjuvant setting [17].

The established neoadjuvant therapies in HER2 positive breast cancer are the AC-THP or TCbHP regimens. However, a recently published study showed that anthracycline combinations do not improve the pCR rates nor survival outcomes [18, 19]. The TRYPHAENA trail also demonstrated similar efficacy for anthracycline-free versus anthracycline-containing regimens [20], while anthracycline-containing regimens had significantly reduced cardiac safety [21]. Therefore, in clinical practice, the TCbHP regimen, which include doxcetaxel and carboplatin, has gained popularity in recent years and become the standard treatment. However, carboplatin causes hematological adverse events, such as anemia and thrombocytopenia [2, 22, 23], which can delay neoadjuvant therapy or surgery and affect therapeutic outcomes [24, 25]. A recently publish paper mentioned that as healthcare provider, we must weigh the therapeutic benefits against short-term and long-term risks [26]. Therefore, while pursuing the pCR rate, we should also pay attention to patient treatment compliance and tolerance in the neoadjuvant therapy setting.

To achieve this goal, some clinical trials in HER2 positive early breast cancer are evaluating further therapy de-escalation in selected patients. The WGS ADAPT trail demonstrated feasibility of avoiding overtreatment and individualizing neoadjuvant therapy. In the HR- HER2-positive breast cancer patients, a pCR rate of about 90% with 12 weeks of paclitaxel weekly plus trastuzumab and pertuzumab (THP) [27, 28]. These results suggest that we may be able to omit the carboplatin and still achieve a good pathological complete response. To further assess whether carboplatin can be de-escalated, the ongoing COMPASS HER2 trail is enrolling patients with stage II and III HER2-positive breast cancer being treated with THP neoadjuvant therapy [29]. Additionally, results from the phase III KATHERINE trial showed that using TDM1 for non-pCR patients after neoadjuvant therapy can provide a guarantee for prognosis [30]. The good news is that TDM1 is now available in China and can be covered by medical insurance. Therefore, a de-escalation treatment plan such as omitting carboplatin, may be feasible.

In our present study, we investigated whether there were significant differences in pCR rates among three neoadjuvant chemotherapy regimens: TCbHP, AC-THP and THP. Subgroup analyses demonstrated that there was no significant difference in pCR rate among the neoadjuvant chemotherapy regimens in the population with ER + , PR + , HER2 IHC 2 + , and Ki67 index < 30%. This indicates that for patients with these characteristics, THP can be used as an alternative to the TCbHP regimen, when taking the high incidence of grade 3–4 thrombocytopenia observed with TCbHP compared to THP into account [31]. However, for ER-PR-HER2-positive patients, the neoadjuvant chemotherapy regimen has a great influence on the pCR rates. Table 7 showed that the pCR rate of patients who received TCbHP regimen was 72% (78/108), but the pCR rate significantly decreased to 52% (43/83) using THP therapy.

This means although more than 50% of the patients who using THP regimen achieved pCR, there is still a 20% gap compared with patients who received with TCbHP regimen. The low pCR rate of the THP regimen may be related to the short chemotherapy cycle. The application of six cycles of THP may improve the pCR rate. Six cycles of THP chemotherapy regimen had been proposed by Chinese Society of Clinical Oncology (CSCO) Breast Cancer guidelines (2023) as one of the neoadjuvant treatment options”. Therefore, HER2-positive breast cancers cannot be treated as a homogeneous group using the same neoadjuvant therapy.

To our knowledge, this is the first study in which the pCR of patients with invasive micropapillary carcinoma is explored in a HER2-positive cohort. A previous study showed that invasive micropapillary carcinoma in invasive ductal breast cancer may benefit less from standard adjuvant trastuzumab and chemotherapy than non-invasive micropapillary carcinoma [32]. Consistent with this, our present study showed that patients with invasive micropapillary carcinoma were hardly achieved pCR. This indicates that HER2-positive patients with invasive micropapillary carcinoma may rarely benefit from neoadjuvant chemotherapy with anti-dual HER2 therapy. Currently, there are still no specific treatment for invasive micropapillary carcinoma. Therefore, the results of this study suggest that oncologist should subject these patients to more intensive monitoring of treatment response and consider other anti-HER2 treatments to improve the pCR rates for HER2 positive invasive micropapillary breast cancer patients.

Some limitations should be taken into account when applying our results. Although the predictive ability of this pCR prediction nomogram constructed based on the combined IHC biomarker was acceptable, it was still not sufficiently refined. First, the IHC based subtype may not be reliable enough to reflect the intrinsic subtype. Second, the routine IHC based HER2 definition cannot precisely represent the amplification level of the HER2 gene [33]. Third, the AUC of the nomogram was 0.73. Therefore, it is necessary to incorporate more effective biological indicators screened by whole exome sequencing or RNA-seq to further improve and enhance predictive ability and provide individual precision treatment for HER2-positibve breast cancer.

## Conclusion

In conclusion, our results showed that patients with ER-negative, PR-negative, HER2 3 + and high KI-67 index were more likely to achieve pCR. THP may be used as an alternative to AC-THP or TCbHP in selected HER2-positive patients. In the era of precision medicine, we should select the most suitable treatment strategy by predicting the sensitivity of HER2-positive breast cancer patients to neoadjuvant therapy, aiming to achieve the best therapeutic outcomes while minimizing side effects.

### Supplementary Information

**Additional file 1:** **Table S1.** Baseline clinicopathological characteristics.

**Additional file 2:** **Table S2.** Patient (who received THP regimen) characteristics according to breast pathological complete response.

**Additional file 3:** **Table S3.** Patient (who received THP regimen) characteristics according to total pathological complete response.

**Additional file 4:** **Table S4.** The sensitivity and specificity of the nomogram for pCR.

## Data Availability

All analyzed data are included in this published article and its supplementary information file. The original data are available upon reasonable request to the corresponding author.
