# Supplementary material for: Real-world data on neoadjuvant chemotherapy with dual-anti HER2 therapy in HER2 positive breast cancer
Source: BMC Cancer. 2024 Jan 25;24:134. doi: 10.1186/s12885-024-11871-0 (PMC10811850; doi:10.1186/s12885-024-11871-0)
Supplement: Supplementary file 1 — Additional file 1: Table S1. Baseline clinicopathological characteristics. [file 12885_2024_11871_MOESM1_ESM.docx]

Table S1 Baseline clinicopathological characteristics.

| Characteristic | All (n=353)  Number (%) |
| --- | --- |
| Age (years), mean±SD | 48.94±10.04 |
| Tumor stage |  |
| cT1-2 | 236 (66.9) |
| cT3-4 | 117 (33.1) |
| Lymph node status |  |
| Negative | 58 (16.4) |
| Positive | 295 (83.6) |
| ER status |  |
| Negative | 200 (56.7) |
| Positive | 153 (43.3) |
| PR status |  |
| Negative | 280 (79.3) |
| Positive | 73 (20.7) |
| Ki-67 index |  |
| <30% | 30 (8.5) |
| ≥30% | 323 (91.5) |
| Histological Grade |  |
| I-II | 189(53.5) |
| III | 164(46.5) |
| HER2 status |  |
| 2+ | 56 (15.9) |
| 3+ | 297 (84.1) |
| IMPC |  |
| With | 22 (6.2) |
| Without | 331 (93.8) |
| Chemotherapy regimens |  |
| THP*4 | 103 (29.2) |
| TCbHP*6 | 213 (60.3) |
| AC*4-THP*4 | 37 (10.5) |
