# Supplementary material for: Real-world data on neoadjuvant chemotherapy with dual-anti HER2 therapy in HER2 positive breast cancer
Source: BMC Cancer. 2024 Jan 25;24:134. doi: 10.1186/s12885-024-11871-0 (PMC10811850; doi:10.1186/s12885-024-11871-0)
Supplement: Supplementary file 2 — Additional file 2: Table S2. Patient (who received THP regimen) characteristics according to breast pathological complete response. [file 12885_2024_11871_MOESM2_ESM.docx]

Table S2 Patient (who received THP regimen) characteristics according to breast pathological complete response.

|  | Non-pCR (n=49) | pCR  (n=54) | All  (n=103) | *P*-value |
| --- | --- | --- | --- | --- |
| Age (years), mean±SD | 52.32±10.80 | 49.22±10.49 | 51.29±10.54 | 0.549 |
| Tumor stage |  |  |  |  |
| cT1-2 | 33 | 37 | 70 | 0.899 |
| cT3-4 | 16 | 17 | 33 |  |
| Lymph node status |  |  |  |  |
| Negative | 9 | 9 | 18 | 0.820 |
| Positive | 40 | 45 | 95 |  |
| ER status |  |  |  |  |
| Negative | 34 | 47 | 81 | **0.029** |
| Positive | 15 | 7 | 22 |  |
| PR status |  |  |  |  |
| Negative | 38 | 51 | 89 | **0.012** |
| Positive | 11 | 3 | 14 |  |
| Histological Grade |  |  |  |  |
| I-II | 27 | 17 | 44 |  |
| III | 22 | 37 | 59 | **0.016** |
| Ki-67 index |  |  |  |  |
| <30% | 2 | 5 | 7 | 0.297 |
| ≥30% | 47 | 49 | 96 |  |
| HER2 status |  |  |  |  |
| 2+ | 9 | 4 | 13 | 0.092 |
| 3+ | 40 | 50 | 90 |  |
| IMPC |  |  |  |  |
| With | 7 | 0 | 7 | **0.004** |
| Without | 42 | 54 | 96 |  |
