# Supplementary material for: Real-world data on neoadjuvant chemotherapy with dual-anti HER2 therapy in HER2 positive breast cancer
Source: BMC Cancer. 2024 Jan 25;24:134. doi: 10.1186/s12885-024-11871-0 (PMC10811850; doi:10.1186/s12885-024-11871-0)
Supplement: Supplementary file 3 — Additional file 3: Table S3. Patient (who received THP regimen) characteristics according to total pathological complete response. [file 12885_2024_11871_MOESM3_ESM.docx]

Table S3 Patient (who received THP regimen) characteristics according to total pathological complete response

|  | Non-pCR (n=57) | pCR  (n=46) | All  (n=103) | *P*-value |
| --- | --- | --- | --- | --- |
| Age (years), mean±SD | 52.28±10.48 | 49.91±10.27 | 51.29±10.54 | 0.740 |
| Tumor stage |  |  |  |  |
| cT1-2 | 39 | 31 | 70 | 0.911 |
| cT3-4 | 18 | 15 | 33 |  |
| Lymph node status |  |  |  |  |
| Negative | 9 | 9 | 18 | 0.616 |
| Positive | 48 | 37 | 85 |  |
| ER status |  |  |  |  |
| Negative | 40 | 41 | 81 | **0.020** |
| Positive | 17 | 5 | 22 |  |
| PR status |  |  |  |  |
| Negative | 44 | 45 | 89 | **0.002** |
| Positive | 13 | 1 | 14 |  |
| Histological Grade |  |  |  |  |
| I-II | 29 | 15 | 44 |  |
| III | 28 | 31 | 59 | 0.062 |
| Ki-67 index |  |  |  |  |
| <30% | 3 | 4 | 7 | 0.491 |
| ≥30% | 54 | 42 | 96 |  |
| HER2 status |  |  |  |  |
| 2+ | 10 | 3 | 13 | 0.092 |
| 3+ | 47 | 43 | 90 |  |
| IMPC |  |  |  |  |
| With | 7 | 0 | 7 | **0.014** |
| Without | 50 | 46 | 96 |  |
