# Supplementary material for: Real-world data on neoadjuvant chemotherapy with dual-anti HER2 therapy in HER2 positive breast cancer
Source: BMC Cancer. 2024 Jan 25;24:134. doi: 10.1186/s12885-024-11871-0 (PMC10811850; doi:10.1186/s12885-024-11871-0)
Supplement: Supplementary file 4 — Additional file 4: Table S4. The sensitivity and specificity of the nomogram for pCR. [file 12885_2024_11871_MOESM4_ESM.docx]

Table S4 The sensitivity and specificity of the nomogram for pCR

| Predictive result | True result | | Total |
| --- | --- | --- | --- |
|  | pCR | Non-pCR |  |
| pCR | 133 | 45 | 178 |
| Non-pCR | 52 | 123 | 175 |
| Total | 185 | 168 | 353 |

(Sensitivity)：71.89%, (Specificity)：73.21%, (Positive predictive value)：74.72%, (Negative predictive value)：70.29%, (Correction rate)：72.52%
